# Contact Breast Injuries Among Female Athletes: A Systematic Review

**DOI:** 10.1007/s40279-024-02027-y

**Published:** 2024-05-01

**Authors:** Kilian Bibby, Ian C. Kenny, Róisín Cahalan, Helen Purtill, Tom M. Comyns

**Affiliations:** 1 Department of Physical Education and Sport Sciences, University of Limerick, Limerick, Ireland; 2 Sport and Human Performance Research Centre, University of Limerick, Limerick, Ireland; 3 Health Research Institute, University of Limerick, Limerick, Ireland; 4 School of Allied Health, University of Limerick, Limerick, Ireland; 5 Physical Activity for Health Research Cluster, University of Limerick, Limerick, Ireland; 6 Department of Mathematics and Statistics, University of Limerick, Limerick, Ireland; 7 Lero, The Science Foundation Ireland Centre for Software Research, University of Limerick, Limerick, Ireland

## Abstract

**Background:**

Robust surveillance of injury aetiology and epidemiology is recognised as fundamental for effective injury reduction and management programmes. However, while sex-specific differences in injury type and nature are noted in the literature, it is unclear if these are reflected in surveillance practices, and how the athlete is affected.

**Objective:**

Therefore, this study aimed to systematically review contact breast injuries (CBIs) among adult female athletes.

**Methods:**

The following databases were searched: PubMed, EMBASE, SPORTDiscus including MEDLINE, Web of Science and Scopus. The literature search was conducted in May 2023 and the search was limited to articles in the English and German language. Studies including female athletes, aged 18 years and above, in any sports (team or individual) at any level (amateur, semi-professional and professional), where an occurrence of CBI was documented were included. Studies were included irrespective of their investigated timeframes (e.g. the whole career, one or multiple seasons). Findings were categorised (e.g. sport, level of competition and investigated timeframe of the study) to enable possible comparisons. Case studies were excluded due to the non-generalisability of findings.

**Results:**

Of the six studies included, rugby codes (rugby union, rugby league and rugby sevens) had the highest occurrence rate (62.0%) of CBIs among eight different investigated sports (rugby codes 62.0%, softball 59.5%, Australian Football League (AFL) 51.0%, water polo 50.0%, soccer 46.7%, basketball 27.6–48.8%, volleyball 34.6%, boxing 0.0%). Between 25.6% and 62.0% of participants reported incurring a CBI and between 0.0% and 42.9% of CBIs were reported to a medical professional or support staff. The reported treatment rate for CBIs ranged between 0.0% and 2.1%, The main mechanisms for CBIs (where reported) were contact with another athlete (AFL 37.6%, rugby codes 56%) the ball (AFL 31.6%, rugby codes 25.5%) and the ground (AFL 6.6%, rugby codes 22%). Between 18.2% and 48% of the participants reported that CBIs negatively affected their performance. Risk factors increasing CBIs were positional differences, larger breast size and higher body mass index (BMI). In-season injury data collection and surveillance supported through education of both players and medical staff were identified to be of relevance for future CBI prevention. None of the studies reported incidence rate.

**Conclusion:**

Despite the frequent occurrence of CBIs among female athletes, reporting and treatment remains low. Awareness and education of all stakeholders are fundamental to ensuring better breast safety in female sport. Identifying the mechanics, severity and risk factors of CBIs through thorough injury surveillance must be a focus of further research.

**Registration:**

The study was preregistered on Open Science Framework (OSF).

## Key Points

**Table:** 

This review investigated the occurrence of contact breast injuries among female athletes, which negatively affect performance but are underreported across all sports we examined.	
Players and medical staff need to be educated regarding the existence of breast injuries to help develop the necessary environment for reporting.	
To simplify injury data collection and improve data quality, a standardised taxonomy for female-specific injuries is needed.	

## Introduction

Global sports participation among females has been growing exponentially [1]. Contrary to this development, only a small portion of research includes or solely focuses on female athletes [2–4]. It is also a common practice in sports to apply processes used in injury surveillance systems that have been developed for male performance settings to female athletes [3, 5] without considering the potential impact on the unique physiological, biomechanical and anatomical characteristics of females [5]. The absence of tailored injury surveillance and interventions specific to female athletes may lead to a failure to optimise strategies to mitigate against future injuries and performance-reducing factors, leading to long-term complications [5]. A contact breast injury (CBI) occurs when the breast is struck with blunt force [6, 7]. Collisions or contact with opponents, the ground, or playing equipment are all potential mechanisms of CBIs [8–10]. Due to its position and anatomy, the female breast is particularly exposed in contact sport [11]. Traumatic breast injuries can cause future complications such as fat necrosis, which may be misdiagnosed as breast cancer [12], mastitis leading to breast abscess [13] or Mondor disease [14]. In general, traumatic soft tissue injuries can cause a pseudoaneurysm mimicking a soft tissue tumour [15], sensory disturbances such as neuropraxia [16] and more widely local swelling, tenderness, pain and decreased sports performance [17].

However, CBIs are an under-reported injury in female athletes, and little is documented about this phenomenon in the current literature [18, 19]; despite ongoing international research, we do not yet have evidence to indicate the state of play on this topic. Depending on the specific sport, injuries may have different mechanisms, locations and occurrence rates. Injury surveillance, documentation and analysis play a vital part in injury reduction [20]. Current literature indicates that prospective injury surveillance is superior to retrospective reporting [21, 22], while use of medical support staff for recording injuries is more valid than using coaching staff [20] or self-reporting [23].

Therefore, the data synthesising process in this study aims to provide general guidance and feedback to female athletes, their support staff and the literature regarding CBIs. A further aim of this review was to identify the occurrence, mechanism and impact of CBIs on female athletes in addition to consideration of recording and treatment practices, and any factors predisposing athletes to CBIs.

## Methods

A review protocol was written and registered via the Open Science Framework (OSF) [24]. Following registration, a systematic search was conducted including any study that provided epidemiological information on the occurrence of CBIs among the population of adult female athletes in any sport. The systematic review was conducted in accordance with the 2020 updated PRISMA guideline for reporting systematic reviews [25].

### Searches

The search strategy aimed to locate both published studies and grey literature such as theses or dissertations. An initial limited search identifying articles on the topic was undertaken on PubMed and Google Scholar. PubMed, EMBASE, SPORTDiscus including MEDLINE with full text, Web of Science and Scopus were searched for title and abstract with the developed search strategy using the Population, Exposure and Outcome (PEO) framework (Table 1).

**Table 1:** Population, exposure and outcome (PEO) framework

PEO framework	Search terms
P: Participants/population	“female” or “women” or “woman” or “females”
E: Exposure	“sport*” or “athlet*”
O: Outcome	“breast” and “injury” or “injuries”

The exact search syntax, including all terms, applicable truncations and Boolean operators, were adapted for each search engine (Table 2).

**Table 2:** Search syntax

Search engine	Exact search syntax	Results
PubMed	((sport*[Title/Abstract]) OR (athlet*[Title/Abstract])) AND ((female[Title/Abstract]) OR (women[Title/Abstract]) OR (woman[Title/Abstract]) OR (females[Title/Abstract])) AND (breast[Title/Abstract]) AND ((injury[Title/Abstract]) OR (injuries[Title/Abstract]))	29
SPORTDiscus and MEDLINE	S13: S9 AND S10 AND S11 AND S12 S12: S7 OR S8 S11: S5 OR S6 S10: S3 OR S4 S9: S1 OR S2 S8: TI injur* S7: AB injur* S6: TI breast S5: AB breast S4: TI female or women or woman or females S3: AB female or women or woman or females S2: TI sport* or athlet* S1: AB sport* or athlet*	49
Scopus	( ABS ( sport*) OR TITLE ( sport*) OR ABS ( athlet*) OR TITLE ( athlet*) AND ABS ( women) OR TITLE ( women) OR ABS ( woman) OR TITLE ( woman) OR ABS ( female*) OR TITLE ( female*) AND ABS ( breast) OR TITLE ( breast) AND ABS ( injur*) OR TITLE ( injur*))	36
Web of Science	(((((((AB = (Sport*)) OR AB = (Athlet*)) AND AB = (Female)) OR AB = (Women)) OR AB = (Woman)) OR AB = (Females)) AND AB = (Breast)) AND AB = (Injur*) (Web of Science Categories: Sport Sciences or Rehabilitation)	28
	(((((((TI = (Sport*)) OR TI = (Athlet*)) AND TI = (Female)) OR TI = (Women)) OR TI = (Woman)) OR TI = (Females)) AND TI = (Breast)) AND TI = (Injur*) (Web of Science Categories: Sport Sciences or Rehabilitation)	6
Embase	(sport:ab,ti OR sports:ab,ti OR athlete:ab,ti OR athletes:ab,ti) AND (female:ab,ti OR females:ab,ti OR women:ab,ti OR woman:ab,ti) AND breast:ab,ti AND (injury:ab,ti OR injuries:ab,ti)	26

We initially identified 174 articles. To detect additional studies, the references list of studies of relevance were screened. Authors of relevant registered study protocols and abstracts were also contacted to identify additional studies in print for potential inclusion.

### Study Inclusion and Exclusion Criteria

The population of interest was female athletes aged 18 and over who had suffered a CBI in any sport. Case studies were excluded due to lack of generalisability. Studies were also excluded if the recorded breast injury was not contact-related (e.g. friction related: chafing, runners- or bicycle nipple [26–28]). Studies were included irrespective of their investigated timeframes (investigations over multiple years or seasons, single-season studies or cross-sectional studies). Studies published in the English and German languages were included.

Following the search, all identified studies were collated and uploaded into Zotero [29] and duplicates were removed. Titles and abstracts were screened by two independent reviewers (KB and RC) and the full texts of appropriate studies were screened by these reviewers against agreed-upon inclusion criteria. Disagreements were adjudicated by an additional reviewer (IK). The study selection process is presented in a Preferred Reporting Items for Systematic Reviews and Meta-Analyses (PRISMA) flow diagram.

### Study Quality Assessment

The Joanna Briggs Institute (JBI) Critical Appraisal Checklist for Analytical Cross-Sectional Studies [30] was used. The critical appraisal including analysis of bias was carried out by two independent reviewers (KB and IK) and disagreements that occurred during this process were adjudicated by an additional investigator (TC). The assessment for the risk of bias was based on the percentile positive (yes) answers of the JBI checklist; ≥ 49.9% = high, 50.0%–74.9% = moderate, ≤ 75.0% = low.

### Data Extraction Strategy

Relevant data were extracted from studies remaining after the screening process. Extracted data included details of participants, study design, concept, context and key findings (Table 3). A statistician (HP) was consulted regarding the possibility of a meta-analysis. Due to differences in objectives and outcome measures across the studies producing substantial heterogeneity, a systematic review only was performed.

**Table 3:** Study characteristics and data extraction

Study	Year	Type of research	Country of data origin	Investigated timeframe	Level of competition	Investigated sport	Number of participants	Occurrence of CBIs^a^
Brisbine et al. [8]	2019	Cross-sectional	Australia	Current career	Elite	Contact/combat sport	*n* = 91	44.4%
						Non-contact sport	*n* = 413	25.6%
Brisbine et al. [9]	2020	Cross-sectional	Australia	Current career	Sub-elite *n* = 193 Elite = 104	AFL	*n* = 125	51.0%
						Rugby Union	*n* = 58	62.0%^b^
						Rugby League	*n* = 68	
						Rugby Sevens	*n* = 46	
Massimiliano et al. [31]	2011	Longitudinal study	Italy	6 years, 8 months	Amateur	Boxing	*n* = 61	0.0%
Smith et al. [32]	2018	Cross-sectional	USA	College career	College	Softball	*n* = 37	59.5%
						Volleyball	*n* = 26	34.6%
						Basketball	*n* = 41	48.8%
						Soccer	*n* = 90	46.7%
Smith et al. [10]	2022	Cross-sectional	USA	Current career	Professional	Water polo	*n* = 16	50.0%
Smith et al. [33]	2023	Cross-sectional	France	Current career	Professional	Basketball	*n* = 58	27.6%

*AFL* Australian Football League

^a^Describes the percentage of participants that stated incurring a contact breast injury (CBI) during the investigated timeframe

^b^Occurrence of contact breast injury (CBI) in three rugby codes (rugby union, rugby sevens and rugby league) [9]

## Results

**Fig. 1:**
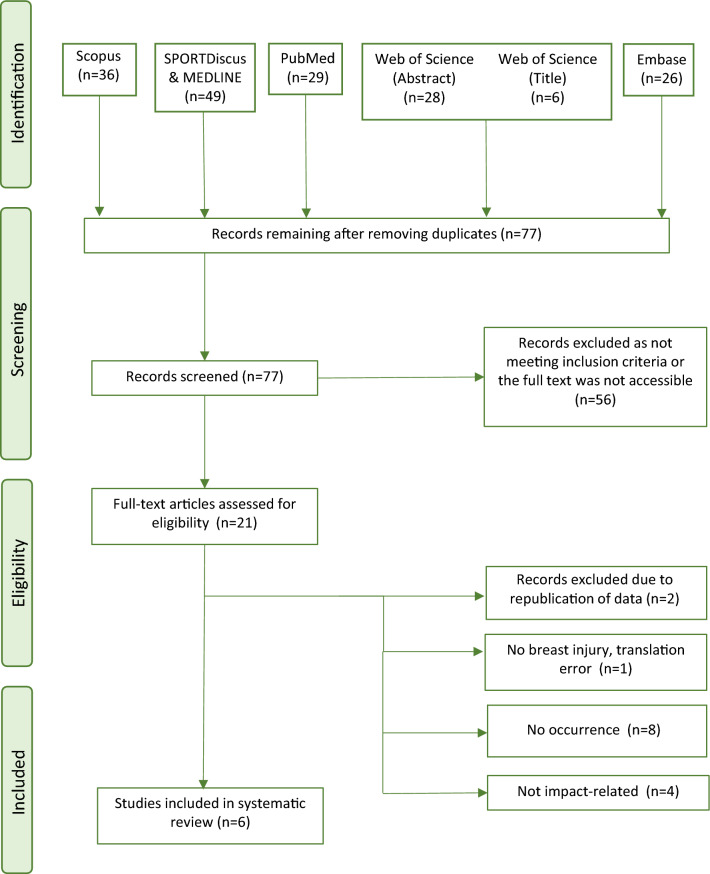
PRISMA flowchart

The preliminary search revealed 77 unique results, of which six were included in this review; reasons for exclusion are outlined in Fig. 1.

### Study Quality Assessment

The results of the critical appraisal process via the JBI Critical Appraisal Checklist for Analytical Cross-Sectional Studies [30] are shown in Table 4.

**Table 4:** Critical appraisal and risk of bias

	JBI critical appraisal checklist for analytical cross-sectional studies [30]									
Study	Q1	Q2	Q3	Q4	Q5	Q6	Q7	Q8	%Yes	Bias
Brisbine et al. [8]	Yes	Yes	Yes	Unclear	Yes	Yes	Unclear	Yes	75.0	Low
Brisbine et al. [9]	Yes	Yes	Yes	Unclear	Yes	Yes	Unclear	Yes	75.0	Low
Massimiliano et al. [31]	Yes	Yes	Yes	Yes	No	No	Yes	Unclear	62.5	Moderate
Smith et al. [32]	Yes	Yes	Yes	Unclear	No	No	Unclear	Yes	50.0	Moderate
Smith et al. [10]	Yes	Yes	Yes	Unclear	No	No	Unclear	Yes	50.0	Moderate
Smith et al. [33]	Unclear	Yes	Yes	Unclear	No	No	Unclear	Yes	37.5	High

Question description: Q1. Clear inclusion criteria; Q2. Detailed subject and setting description; Q3. Valid and reliable exposure measurement; Q4. Objective, standard criteria for condition measurement; Q5. Identifying confounding factors; Q6. Strategies to deal with confounding factors; Q7. Valid and reliable outcome measurement; Q8. Use of appropriate statistical analysis

#### Risk of Bias

Of included studies, two contained a low risk of bias [8, 9], three showed a moderate risk [10, 31, 32] with a high risk of bias identified in one study [33].

### Study Characteristics

Data from eight different sports in four different countries were included in the study (Table 3).

#### Contact Breast Injury (CBI) Prevalence, Mechanism and Protective Equipment

The prevalence of sustaining a CBI was only recorded by two included studies [10, 33]. In French basketball players, 35.7% of participants recorded sustaining at least three CBIs [33], while 62.5% of US American water polo players incurred six or more CBIs on different occasions during their careers [10].

Three studies explored mechanism of CBIs, of which two identified a direct blow from another athlete, equipment or the ground as factors for CBIs [8, 10]. In the third study, comparing rugby codes and Australian Football League (AFL), contact with another player was the leading mechanism for CBIs (AFL 37.6%, rugby codes 56%), followed by contact with the ball (AFL 31.6%, rugby codes 25.5%), and the ground (AFL 6.6%, rugby codes 22%) [9].

None of the female professional French basketball players nor any of the US American water polo players wore protective equipment [10, 33]. For college athletes, 2.1% specified wearing additional breast protective equipment next to their normal breast support [32]. In a multisport comparison, only 3.0% of participants who suffered a breast injury mentioned wearing additional breast padding. In over 90% of those cases, the padding was either mandatory or actively encouraged [8]. The negative impact of CBIs on the performance of participants was discussed in four of the included studies, and affected 18.2% [32], 21.0% [8], 28.6% [33] and 48.0% [9] of participants, respectively. Adverse impacts on the performance of participants included movement modifications [8, 9], pain [32] and time loss [33] due to a CBI.

#### Risk Factors for CBIs

Two studies [8, 9] explored risk factors for CBIs and identified that athletes who reported breast injuries had, on average, larger breasts (greater surface area for potential injury) and a higher BMI when compared with participants who did not report a CBI [8]. Another factor was positional differences, with AFL midfielders and forwards at a higher risk of incurring a CBI compared with backs. In rugby, there was no significant association found between playing position and the risk of incurring a CBI [9].

#### Reporting and Treatment Rate of CBIs

Reporting rates of CBIs to a coach, medical or other support staff varied from 0.0% [10] to 9.6% [32], 10.0% [8] and 42.9% [33] in the reviewed studies. Treatment rates of reported CBIs, where reported, differed, from 0.0% [10] to 2.1% [32] and 50.0% [33].

## Discussion

This systematic review has identified that between 25.6% [8] and 62.0% [9] of female athletes experienced one or more CBI, with 18.2% [32] to 48.0% [9] of these injuries having a negative impact on their performance. Contact with another athlete was the leading mechanism for CBIs [8–10], while contact with non-player items was also associated with CBIs. There is limited research on CBIs, and the quality of studies is variable. Apart from Smith et al. (2023), who documented severity in terms of time loss [33], none of the remaining five studies provided information on the incidence rate or severity of CBIs, as recommended by the International Olympic Committee (IOC) [34]. Five studies were designed as player self-report of injury, which can lead to errors of recall and reporting. The current literature suggests that reporting and treatment of CBIs is extremely low overall.

### CBI Prevalence and Inter/Intra-Sport Comparison

When comparing the documented prevalence of CBIs, there are indications of clear differences between sports. In US water polo players, 62.5% of participants had six or more CBIs compared with at least three CBIs among 35.7% of professional French basketball players [10, 33]. This review only offered one intra-sport comparison, identifying that female US college basketball players were at greater risk (48.8% [32]) of sustaining a CBI than French professional basketball players (27.6% [33]). However, the utility of this comparison is mitigated when one considers the status of the players, namely professional versus collegiate athletes. The heterogeneity of other included sports in this review made it impossible to generate an inter-sport comparison of CBI prevalence.

The importance of future CBI research can be seen when comparing compiled prevalence of CBIs (between 25.6 and 62.0%) with other female injury epidemiology research; for example, concussion (46.6–78.3% in contact sports [35] and 52.5% among Gaelic football [36]), stress fractures (14.0% within multiple sports [23]) and anterior cruciate ligament injuries (28.2% in alpine skiers [37]).

### Risk and Impact of CBIs and Protective Equipment

The identified risk factors for sustaining a CBI included a higher BMI, larger breasts and field or court positional differences [8, 9]. A higher BMI has previously been shown to increase the risk of injury in females [38], as has the risk of injury associated with positional differences in sports such as soccer [39] and rugby union [40]. While BMI, and to an extent playing position, are modifiable, identifying unmodifiable risk factors is particularly important as this can directly guide injury prevention strategies towards the population with the most persistent need [41]. Injury prevention can be supported by identifying risk factors leading to injury [42], therefore greater awareness and moderating actions to minimise the risk of CBIs are advocated.

The negative impact of CBIs on performance was mentioned by athletes in two included studies. These consisted mainly of sport averse movement modification to prevent the reoccurrence of previously experienced painful breast injuries [8, 9]. Sport averse movement modifications, or movement compensations, have the potential to cause joint pain and osteoarthritis [43] as well as extra tissue stress leading to additional anatomical damage [44]. While pain due to breast injury adversely affected female college athletes, it is noteworthy that all these athletes continued to compete [32]. In comparison, 75% of French professional basketball players who had a CBI reported that this resulted in time loss and absence from playing [33]. The diverse and overwhelmingly negative implications of time-loss injuries have been widely reported and include league table positioning, competition outcomes and general success [45, 46]. As the current paper highlights, performance decrements through CBIs are not clearly empirically examined, therefore further studies focussing on the implications of CBIs are required.

Investigation of the utilisation of protective equipment indicated that most athletes did not wear anything in addition to their normal breast support (college athletes between 0.0 and 2.7% [32]). Of the athletes that had received CBIs in a multi-sport study, only 3% reported wearing additional breast padding [8]. Other studies that asked female players about additional breast protective equipment stated that it is not being worn [10, 33]. In boxing, breast protective equipment is either mandatory or highly recommended [8, 31]. Although perceived to be protective, breast protective equipment among female contact football players was reported to be not commonly worn, and the reasons identified were the lack of awareness of existence, discomfort and poor fit [47]. The role and possible benefits of protective equipment for female athletes requires focused investigation [11].

### Reporting and Treatment Rate of CBIs

The 2020 updated Orchard Sport Injury and Illness Classification System (OSIICS) [48] included breast hematoma/trauma in injury and illness coding systems [5]. Additionally, a current recommendation endorses the adoption by researchers of reporting consensus from the IOC on methods for recording and reporting epidemiological data on injury and illness in female athlete health domains [11]. Nevertheless, despite this recent recognition of one of many breast health issues and other female-specific health risks, CBIs remain largely undocumented with restricted data quality [5].

In the current study, the rate of CBIs reported ranged between 0.0 [10] and 42.9% [33]. Non-reporting in some studies did not necessarily mean that there were no injuries, but likely that injuries were not reported by the female athletes. In the current literature, reasons for under- or nonreporting of injuries included intrinsic and extrinsic pressures, fear of judgment by coaches and/or teammates, non-recognition of symptoms, or belief that the injury is not severe enough [49–52]. Research indicates that athletes appear to be more likely to report an injury to an injury recorder of the same sex [53] and in supportive environments [54]. Improving athletes’ knowledge [55] and long-term complications [56] associated with CBIs, while creating a trusting and supportive environment [57], should positively affect the reporting rate of CBIs. Five of the six studies [8–10, 32, 33] specifically identify the creation of awareness and education programmes regarding the existence of CBIs as areas for future research.

From a medical standpoint, further standardised taxonomy for female-specific injury identification is needed as it could lead to improved reporting methods [5, 11]. The low recorded treatment rate (0.0–50.0%) when a CBI is actually reported may indicate a lack of awareness and knowledge on the part of healthcare professionals and the wider backroom staff. It must be mentioned that only three studies discussed treatment rates [10, 32, 33] and participants in the other included study cohorts might have received treatment without it being reported.

### Limitations

The small number of studies that examined CBIs in sport and fulfilled the inclusion criteria limited the systematic review to just six papers, which could not be considered for meta-analysis due to their differences in objectives and outcome measures. Apart from Massimiliano et al. (2011), who used independent medical professionals for prospective injury recording [31], all other studies used an anonymous questionnaire to ask the female players retrospectively about their experience with breast injuries. Due to the sensitive topic, the choice of an anonymous questionnaire is understandable [58], but may compromise reliability and validity when compared with a medical examination [59]. Apart from two studies involving basketball, no intra-sport comparison could be performed as all other investigated sports were of different types.

## Conclusion

From the limited research available, CBIs are a potentially serious and underreported issue in female sport. This review study identified that apart from boxing, all included sports showed a CBI occurrence of at least 25.6% and as high as 62.0%. The most documented mechanism for a CBI among 37.6% of AFL and 56.0% of rugby athletes was contact with another player. CBIs may have serious consequences for athletes, and responsible reporting, treatment and education are a priority.

There is a demand for in-depth knowledge and research about CBIs to support the ongoing, ubiquitous rise of female sport participation around the world, but focussed healthcare for these athletes is lagging. Awareness and education are lacking, with research required to explore large cohorts while abiding by stringent reporting mechanisms largely absent. The low number of studies investigating a small range of sports shows the need for research on CBIs.

Identifying the mechanics, incidence rate and severity of CBIs must be one of the prime foci in further large(r) scale research. Future investigations should also investigate the potential role of protective equipment and the holistic impact of CBIs on participants.

There is a need for structured in-season injury data collection and surveillance, including CBIs. Education of both players and medical support staff to create awareness and the necessary open environment to report CBIs is required. To simplify injury data collection and guarantee data quality, a standardised taxonomy for female-specific injuries is needed. Only through knowledge of all stakeholders is there the possibility of systematic data collection, monitoring and ultimately reduction of CBIs.

## Data Availability

There is no dataset publicly available for this study.

## Declarations

### Organisation/Affiliation

University of Limerick, Ireland | University of Limerick | University of Limerick (ul.ie).

Sport and Human Performance Research Centre, University of Limerick |www.ul.ie/shprc. Health Research Institute, University of Limerick | www.ul.ie/hri. Department of Mathematics and Statistics, University of Limerick | Department of Mathematics and Statistics | University of Limerick (ul.ie).
